# What are the underlying factors for the severity of cyclic mastalgia?

**DOI:** 10.1371/journal.pone.0330468

**Published:** 2025-10-14

**Authors:** Soheila Nazarpour, Masoumeh Simbar, Zahra Kiani, Neda Khalaji, Mobina Khorrami Khargh

**Affiliations:** 1 Department of Midwifery, Chalous Branch, Islamic Azad University, Chalous, Iran; 2 Midwifery and Reproductive Health Research Center, Shahid Beheshti University of Medical Sciences, Tehran, Iran; 3 Department of Midwifery and Reproductive Health, School of Nursing and Midwifery, Shahid Beheshti University of Medical Sciences, Tehran, Iran; Mazandaran University of Medical Sciences, IRAN, ISLAMIC REPUBLIC OF

## Abstract

**Background:**

Cyclic Mastalgia (CM) is the most common breast complaint among reproductive-aged women and can affect their quality of life. The exact etiology is not completely understood, but several factors are suggested to be effective. This study aimed to assess the relationship of some possible related factors with CM.

**Methods:**

This cross-sectional study was conducted on 335 female students with severe cyclic Mastalgia. They were selected by multi-stage sampling method from dormitories of Shahid Beheshti University of Medical Sciences in Tehran-Iran. Data was collected using an online questionnaire including the Beck’s Depression Inventory, the Spielberger Anxiety Inventory, the Fisher Body-Image Questionnaire, the Cardiff Breast Pain Chart (NDBP), and a socio-demographic questionnaire. The data were analyzed by SPSS 29 and using Spearman, Mann-Whitney, Kruskal-Wallis, and multiple linear regression tests.

**Results:**

The average age of the participants was 25.61 ± 5.92 years with a Cardiff score of 31.76 ± 4.33 (mean ± SD). The mean scores for body image, anxiety, and depression were 159.33 (±37.62), 43.35 (±10.32), and 10.80 (±9.79), respectively. Beck’s depression score had a significant positive correlation with the severity of CM (P = 0.035). A significant positive correlation was also found between smoking and the severity of CM (P = 0.035, *r* = 0.115). There were significant positive correlations between the severity of CM with the duration of the menstrual cycle (P < 0.001) and menstruation (P = 0.001). There were no significant relationships between the severity of CM with other variables. The multiple linear regression test demonstrated that depression (P = 0.014) and duration of the menstrual cycle (P = 0.001) are predictors of severity of CM.

**Conclusion:**

Depression and duration of menstrual cycle are potential predictors of the severity of CM. Promotion of Women’s menstrual and mental health as well as prevention of high-risk habits such as smoking should be considered for the severity of CM and improving the quality of life.

## Introduction

Mastalgia (mastodynia) is the most common breast complaint experienced by women of reproductive age [1,2], with a prevalence ranging from 41 to 79 percent [3]. Approximately two-thirds of women experience this pain during their reproductive life, and about 10 percent seek treatment due to its severity impacting daily activities or fear of underlying pathology such as breast cancer [2–5]. Nevertheless, mastalgia remains challenging to manage due to its subjective nature and multifactorial etiology [6].

Among Iranian women, cyclic mastalgia (CM) demonstrates considerable clinical significance. Shobeiri et al. reported that 60 percent of participants experienced CM, with 37.5 percent describing their symptoms as moderate-to-severe [7]. Further supporting these findings, Vaziri et al. demonstrated that CM constitutes the majority (81 percent) of all mastalgia cases [8]. The condition most commonly manifests during the third decade of life [9].

Clinically, mastalgia is classified as cyclic, non-cyclic, or extramammary [2,10–12]. Cyclic mastalgia (CM), the most prevalent type [3] and the focus of this study, is hormonally mediated. It is characterized by bilateral, cyclical pain and swelling that typically worsen 1–2 weeks before menses and subsides after its onset, primarily affecting women aged 20–40 [2,13]. In contrast, non-cyclic mastalgia is often unilateral and not linked to the menstrual cycle, while extramammary pain originates from extra-breast structures but is perceived in the breast [11,12].

Mastalgia significantly impairs daily activities, sleep, and sexual function [14,15], thereby reducing health-related quality of life [16]. The severity of pain and fear of breast cancer further exacerbate this decline [17] and are associated with an increased prevalence of anxiety, depression, and fatigue [18]. Furthermore, the perception of mastalgia and subsequent health-seeking behaviors vary across cultural contexts [4].

Effective management of mastalgia requires a well understanding of its related factors [4]. The proposed risk factors for mastalgia can be categorized into several thematic groups. Hormonal fluctuations are considered primary contributors, especially in cyclic mastalgia [3,6]. Lifestyle factors, including diet (e.g., high caffeine, fatty foods, salt) [2–4,19], smoking [2,3,12,20,21], alcohol consumption [21], physical activity levels [22], and obesity [4,14,21,23], have been frequently studied, yet findings remain markedly inconsistent across different populations. Furthermore, psychosocial factors such as anxiety, depression, and stress demonstrate a significant association with mastalgia severity, although a causal relationship is still debated [2–4,7,16,20,24–27]. Other explored factors include obstetric history [4,21,23], breast characteristics [4,20,21,24], and sociodemographic elements (e.g., employment, education, income) [19,28–30], which have also been significantly associated with mastalgia in various studies. This pervasive lack of consensus (primarily regarding lifestyle and psychosocial factors) underscores the condition’s complex and likely interactive etiology.

Therefore, to bridge this knowledge gap and resolve the existing controversies surrounding the underlying factors for the severity of CM, particularly in a young demographic, this study aimed to investigate the relationship between various potential determinants and the severity of cyclic mastalgia among Iranian female students.

## Methods

### Design of the study

This was an analytical cross-sectional study of 335 female students who were selected by a two-stage random sampling method. The recruitment period for this study was from January 26, 2023 to January 21, 2024. Inclusion criteria were being a female student aged 15–45 years who experienced severe cyclic mastalgia, operationally defined as bilateral breast pain that begins or worsens in 1–2 weeks before menses and subsides shortly after menstruation onset. Exclusion criteria were applied to minimize confounding effects and included: 1) current use of hormonal contraceptives, phytoestrogen-containing herbs (e.g., soy), or antidepressants; 2) history of breast trauma or diagnosed chronic diseases known to influence pain or hormonal balance (e.g., hypertension, diabetes, cancer); 3) recent experience of significant psychological stressor (e.g., loss of a relative within the past 6 months); 4) current pregnancy or breastfeeding; and 5) any self-reported or detected breast abnormality (e.g., lump, wound, mass).

### Sampling method

Sampling was performed using a multi-stage approach. In the first stage, four dormitories affiliated with Shahid Beheshti University of Medical Sciences in Tehran-Iran were randomly selected. In the second stage, a quota sampling strategy was implemented within each selected dormitory to ensure proportional representation based on the total number of resident students in each dormitory. The required sample size was proportionally allocated to each dormitory. Finally, within each quota, participants were randomly selected from the list of eligible residents using the Excel random selection option. Then, the participants were informed about the objectives of the study, and after obtaining the written informed consent the questionnaires were completed electronically by the participants.

### Sample size calculation

The sample size was calculated using the following formula for correlation studies:


$$N\;=\frac{{\left(Z_{\alpha }+\;Z_{\beta }\right)}^{\text{2}}}{C^{\text{2}}}+\;\text{3}=\text{ 304}$$

The parameters for the calculation were defined as follows:

- **α (Type I error rate) = 0.05** (two-tailed)
- **β (Type II error rate) = 0.20** (power = 80%)
- **r (Expected correlation coefficient) = 0.16**, based on the study by Katar & Başer [16]
- **C (Fisher’s Z transformation)** was calculated as:

$$C=\text{0}.\text{5}\;\times \text{ ln }\left(\frac{\text{1}\;+\;r}{\text{1}\;-\;r}\right)=\;\text{0}.\text{161}$$

- **Zα (Standard normal deviate for α) = 1.96**
- **Zβ (Standard normal deviate for β) = 0.84**

The calculated minimum sample size was 304. To account for potential dropouts, the final sample size was increased to 335 participants.

### Tools for data collection

The tools for data collection were (1) a sociodemographic questionnaire, (2) a Beck Depression Inventory, (3) a Spielberger Anxiety Inventory, a Fisher Body-Image Questionnaire, and a Breast Pain Severity Assessment Scale (NDBP) (Cardiff breast pain chart).

#### Nominal days with severe pain score (NDBP) and breast pain assessment scale (Cardiff chart).

Breast pain severity was assessed using the Cardiff Breast Pain Chart [22,26,31]. In the present study, the chart was adapted as a four-point scale including ‘no pain,’ ‘mild to moderate pain,’ ‘severe pain,’ and ‘not applicable’ (for days outside the menstrual cycle). Participants recorded daily pain intensity for two complete menstrual cycles. The severity was quantified using the Nominal Days with Breast Pain (NDBP) score, calculated as:


 [(number of days of mild or moderate pain × 1) + (number of days of severe pain × 2)× 28÷total number of days in a cycle when pain is recorded.

A score >14 indicated severe pain, which served as the primary outcome measure and key inclusion criterion.

The Cardiff chart is a reliable and valid tool. In previous studies, its reliability was reported above 0.8 [32].

#### The second version of Beck depression inventory BDI-II (Beck depression inventory-II).

The Beck Depression Inventory-II (BDI-II) was used to evaluate depressive symptoms. This 21-item self-report tool measures the severity of depressive symptoms across emotional, cognitive, motivational, and physiological domains on a 4-point scale (0–3) [33–35]. Total scores range from 0 to 63, with higher scores indicating more severe depression [36]. The validity, reliability, factor analysis, and cutoff point of this questionnaire have been examined and confirmed by several studies [33,35,37].

#### Spielberger State-Trait Anxiety Inventory (STAI).

The Spielberger State-Trait Anxiety Inventory (STAI) was used to measure trait anxiety. This 40-item self-report instrument consists of two subscales: state anxiety (transient emotional state) and trait anxiety (stable anxiety propensity), each containing 20 items rated on a 4-point Likert scale. The trait anxiety subscale was used in this study to assess participants’ general anxiety levels. Total scores range from 20 to 80, with higher scores indicating greater anxiety severity [38]. The STAI has demonstrated strong psychometric properties, including high internal consistency (Cronbach’s α > 0.89) and test-retest reliability [38–40]. The Persian version of the STAI has been validated in multiple studies and is widely used in Iranian populations [41,42].

#### Fisher Body Image Questionnaire.

The Fisher Body Image Questionnaire was used to assess body image satisfaction. This 46-item self-report instrument measures satisfaction with various body parts on a 5-point scale (1 = very dissatisfied to 5 = very satisfied). Total scores range from 46 to 230, with higher scores indicating greater body satisfaction. The scores lower than 46 indicate no disorder and the scores ≥46 show the disorder [43]. The questionnaire has demonstrated good validity and reliability in previous studies. The Persian version has been validated and shows adequate psychometric properties for use in Iranian populations [44–46].

The scores of all three above-mentioned questionnaires were also calculated to percent by using the following formula: (Score-Min)/(Max-Min)×100.

#### Socio-demographic questionnaire.

This questionnaire included 56 questions about personal and social characteristics, breast and breastfeeding history, medical and family records, nutritional status and habits, physical activities, and menstrual cycle.

### The procedure of the study

After explaining about objectives and procedure of the study and obtaining the written informed consent, the eligible participants were explained about the electronic completion of the questionnaire. The participants should complete and submit the forms after completing the second cycle of the Cardiff chart. The researchers followed up the participants through phone calls.

### Statistical analysis

The data were analyzed using the SPSS statistics software (version 29). First, the normality of the data was tested. Then, based on the results, which showed the non-normal distribution of the dependent variable and most of the independent variables, non-parametric tests were used. The relationships were assessed using the Spearman correlation, Mann-Whitney, and Kruskal-Wallis tests. Stepwise multiple linear regression was used to assess the predictability of variables for mastalgia as the dependent variable. The level of significance was set at *p* less than 0.05.

### Ethics approval

The study was approved by the ethics committee of the Shahid Beheshti University of Medical Sciences, with the code “IR.SBMU.PHARMACY.REC.1401.116” on 2022.08.09. Written informed consents were obtained from the participants. All the study procedures were carried out under the principles in the Declaration of Helsinki 1964 and its amendments later on.

## Results

Three hundred and thirty-five female students with CM living in the dormitory with an average age of 25.61 ± 5.92 years (mean ± SD) participated in the study. The menarche age of these participants was 12.84 ± 1.54 years old. The demographic and social characteristics of participants are shown in Table 1.

**Table 1:** Socio-demographic characteristics of the participants of the study (n = 335).

Variables		Mean±SD/n(%)
Age (year)		25.61 ± 5.92
Menarche (year)		12.84 ± 1.54
BMI (kg/m^2^)		23.27 ± 3.74
Marriage age (if married) (year)		22.50 ± 5.04
Duration of marriage (if married) (year)		7.48 ± 5.66
Spouse’s age (if married) (year)		35.01 ± 6.63
Marital Status	Single	234 (69.9)
	Married	101 (30.1)
Education (Degree)	Associate Diploma	10 (3.0)
	Bachelor’s degree	215 (64.2)
	Master’s degree	59 (17.6)
	Doctorate	51 (15.2)
Education of spouse (if married)	Diploma	13 (12.9)
	Associate Diploma	9 (8.9)
	Bachelor’s degree	49 (48.5)
	Master’s degree	22 (21.8)
	Doctorate	8 (7.9)
Occupation	Employee	105 (31.3)
	Worker	1 (0.3)
	Housewife	21 (6.3)
	Working at home	11 (3.3)
	Student only	197 (58.8)
Spouse occupation (if married)	Employee	55 (54.5)
	Worker	4 (4.0)
	Free work	38 (37.6)
	Retired or unemployed	2 (2.0)
	Student	2 (2.0)
The adequacy of family income	In financial well-being	10 (3.0)
	Sometimes financial problems	67 (20.0)
	Always a financial problem	174 (51.9)
	Complete poverty	84 (25.1)

Two hundred and four (84.5 percent) of the participants have no history of breastfeeding and 109 (32.5 percent) used a tight-fitting bra. Table 2 demonstrates the distribution of the participants by breast and breastfeeding history.

**Table 2:** Distribution of the participants by the breast and breastfeeding history (n = 335).

	Variables	N (%)
The number of children you have breastfed	0	283 (84.5)
	1	10 (9.0)
	2	21 (6.3)
	3	1 (0.3)
Duration of breastfeeding (months)	0	282 (84.2)
	1-12	9 (2.7)
	13-24	28 (8.4)
	25-36	6 (1.8)
	≥37	10 (3.0)
Bra size used	≤ 65	31 (9.3)
	70-75	180 (53.7)
	80-85	117 (34.9)
	≥90	7 (2.1)
Type of bra	Tight bra	109 (32.5)
	Looser bra	226 (67.5)
History of breastfeeding	52 (15.5)	
History of breast surgery	7 (2.1)	
History of benign breast diseases	34 (10.1)	
History of breast cancer	0 (0.0)	
Family history of breast cancer	48 (14.3)	

Table 3 shows the distribution of participants by their nutritional status and habits.

**Table 3:** Distribution of participants by nutritional status and habits of (n = 335).

Variables		N (%)
Smoking (cigarettes/day)	0	327 (97.6)
	1	2 (0.6)
	2	4 (1.2)
	3	2 (0.6)
Hookah (hours/week)	0	323 (96.4)
	1-2	7 (2.1)
	3-4	2 (0.6)
	≥5	3 (0.9)
Coffee and caffeinated drinks (cup/day)	0	165 (49.3)
	1-3	160 (47.8)
	4-5	7 (2.1)
	≥6	3 (0.9)
Chocolate (average package/day)	0	117 (34.9)
	1-3	193 (57.6)
	4-6	23 (6.9)
	7-8	2 (0.6)
Non-alcoholic carbonated drinks (glass/ day)	0	220 (65.7)
	0.5-1	98 (29.3)
	2-3	16 (4.8)
	4	1 (0.3)
Non-alcoholic non-carbonated beverages (glass/ day)	0	245 (73.1)
	0.5-2	77 (23.0)
	3-5	7 (2.1)
	≥6	6 (1.8)
Alcoholic drinks (cups/ day)	0	331 (98.8)
	1	4 (1.2)
Tea (cup/day)	0	45 (13.4)
	1-3	226 (67.5)
	4-6	57 (17.0)
	7-10	7 (2.1)
Fast food (times/week)	0	81 (24.2)
	1-2	213 (63.6)
	3-4	38 (11.3)
	5-6	3 (0.9)
Water (glass/ day)	0	2 (0.6)
	1-4	131 (39.1)
	5-8	182 (54.3)
	9-12	20 (6.0)
Average daily consumption of fat	Low fat	80 (23.9)
	Medium fat	242 (72.2)
	Fatty	13 (3.9)
Average daily consumption of salt	Low salt	88 (26.3)
	Medium salt	219 (65.4)
	Salty	28 (8.4)
Average daily consumption of sugar	Low	166 (49.6)
	Medium	136 (40.6)
	Much	33 (9.9)
Average daily consumption of spices	Low	42 (12.5)
	Medium	215 (64.2)
	Much	78 (23.3)
Average daily consumption of fruits and vegetables	Low	111 (33.1)
	Medium	188 (56.1)
	Much	36 (10.7)
The amount of weight change in the last 5 years	Increase	147 (43.9)
	Decrease	79 (23.6)
	Unchanged	109 (32.5)

Table 4 indicates the distribution of the participants based on the features of the menstrual cycle in the study.

**Table 4:** Distribution of the participants based on the features of the menstrual cycle (n = 335).

Variables		Mean ± SD/n(%)
Menstrual cycle length (days)		28.80 ± 2.86
Duration of menstrual bleeding (days)		6.02 ± 1.89
Regularity of menstrual cycles	Regular	23 (6.9)
	Fairly regular	141 (42.1)
	Irregular	171 (51.0)
Menstrual pain	No	0 (0.0)
	Mild	241 (71.9)
	Moderate	0 (0.0)
	Severe	94 (28.1)
The severity of menstrual bleeding	Low	35 (10.4)
	Medium	248 (74.0)
	Much	52 (15.5)

Table 5 demonstrates the distribution of participants by physical activity and sleep duration.

**Table 5:** Distribution of the participants by their physical activity and sleep duration (n = 335).

Variables		Mean ± SD/n(%)
Physical activities (hours per week)		2.96 ± 4.28
Duration of sleep (hours/24 hours)		7.80 ± 1.45
Regular exercise	Yes	0 (0.0)
	Relatively	301 (89.9)
	No	34 (10.1)
Physical activity during the day	Low	95 (28.4)
	Medium	202 (60.3)
	Much	38 (11.3)

The average scores of CM, body image and its domains, depression, and trait anxiety are shown in Table 6. The results demonstrated Cardiff’s score as 31.76 ± 4.33. The average total score of body image was 159.33 ± 37.62. The lowest and highest scores of dimensions of body image were related to the overall image (58.06 ± 20.35) and upper limbs (65.83 ± 22.86), respectively. There was no body image disorder (Body Image score ≥46) among the participants of the study and all subjects had scores higher than 46. The average scores for anxiety and depression were 43.35 ± 10.32 and 10.80 ± 9.79, respectively. Among the participants, 78.5 percent experienced some degree of anxiety and 44.5 percent some degree of depression.

**Table 6:** The participants’ average scores of cyclic mastalgia, body image, depression, and trait anxiety (n = 335).

Variables	Mean ± SD/ N (%)	
	Score	Score (0–100)
**Cyclic Mastalgia (severe: score >14)**	31.76 ± 4.33	
**Body Image**	**159.33 ± 37.62**	**61.59 ± 20.45**
Head & face	41.93 ± 10.39	62.34 ± 21.64
Upper limbs	36.33 ± 9.15	65.83 ± 22.86
Lower limbs	21.27 ± 5.36	63.62 ± 22.33
Overall	59.80 ± 14.68	58.06 ± 20.35
**Mental Health Condition**		
**Trait Anxiety**	**43.35 ± 10.32**	**38.92 ± 17.21**
Normal (20–34)	72 (21.5)	
Mild (35–45)	131 (39.1)	
Moderate (46–56)	94 (28.1)	
Severe (≥57)	38 (11.3)	
**Depression**	**10.80 ± 9.79**	**17.14 ± 15.53**
Normal (1–10)	186 (55.5)	
Mild Mood (11–16)	68 (20.3)	
Borderline depression (17–20)	25 (7.5)	
Moderate depression (21–30)	41 (12.2)	
Severe depression (31–40)	14 (4.2)	
Extreme depression (≥40)	1 (0.3)	

Spearman’s correlation test demonstrated no significant correlation between the severity of CM with the total score and the scores of all dimensions of body image. There was also no significant correlation between the score of anxiety and the severity of CM. There was a significant positive correlation between Beck’s depression and the severity of CM (P = 0.035) (Table 7).

**Table 7:** Correlations between severity of cyclic mastalgia with the psychological characteristics (n = 335).

Psychological characteristics	Cyclic mastalgia (NDBP)	
	*r*	P
**Body Image**		
Head & face	−0.011	0.838
Upper limbs	−0.002	0.967
Lower limbs	−0.033	0.549
Overall	−0.044	0.425
Total Body Image	−0.032	0.559
**State Anxiety**	0.069	0.206
**Depression**	0.115	**0.035**

Test: Spearman’s rank correlation coefficient.

Spearman’s correlation test did not indicate any significant relationship between the severity of CM with age, menarche age, BMI, and bra size. Also, there were no significant correlations between the severity of CM with the age of the spouse, the age at marriage, and the duration of the marriage.

The Mann-Whitney and Kruskal-Wallis tests indicated no significant differences in the severity of CM in different marital statuses, the occupation of the participant and her spouse, the educational level of the participant and her spouse, and the adequacy of the family’s monthly income. According to the Mann-Whitney test, there was also no significant difference in the severity of CM based on the history of breastfeeding and the type of bra (tight vs. loose).

To assess the relationship between the participants’ nutritional status and habits, the Spearman correlation, and the Mann-Whitney and Kruskal-Wallis tests were used. The results demonstrated a significant positive correlation between daily cigarette smoking and the severity of CM (P = 0.035, *r* = 0.115). The severity of CM had no significant relationship with other nutritional status and habits (including daily use of hookah, coffee and caffeinated drinks, tea, water, non-alcoholic carbonated beverages, non-carbonated non-alcoholic beverages, alcoholic beverages, chocolate, fast food per week, fat, salt, sugar and spices, fruits and vegetables, and the weight change in the last 5 years).

Mann-Whitney test demonstrated the significant differences between the participants who had a history of breast surgery, a history of benign breast diseases, a history of breast malignancy, or a history of breast cancer in the family, with the participants who did not have these histories.

Besides, significant positive correlations were found between the severity of CM with the duration of the menstrual cycle (P < 0.001) and the duration of menstruation (P = 0.001). There were no relationships between CM with menstrual regularity, dysmenorrhea, and the severity of menstrual bleeding (Table 8).

**Table 8:** The relationship between the severity of cyclic mastalgia and the Menstrual pattern (n = 335).

Menstrual pattern	Cyclic mastalgia (NDBP)		
***Spearman’s correlation coefficient***	***r***		***P***
Duration of Menstrual cycle (days)	0.275		**<0.001**
Duration of menstruation (days)	0.183		**0.001**
***Kruskal–Wallis/ Mann-Whitney***	***Mean Rank***		***P***
Regularity of menstrual cycles	Regular	165.02	0.638
	Fairly regular	162.57	
	Irregular	172.87	
Menstrual pain	Mild	169.66	0.615
	Severe	163.74	
The severity of menstrual bleeding	Low	161.84	0.874
	Medium	167.86	
	Much	172.79	

The results demonstrated no significant relationship between the severity of CM with physical activity and sleep duration.

The severity of CM was considered as the dependent variable for the multiple linear regression model. Then, the variables of depression, anxiety, body image, cigarette smoking, duration of menstrual cycle, and bleeding which showed significant relationships with the severity of CM were entered into the model as the independent variables using the Stepwise method. Depression and the duration of the menstrual cycle are shown to be the predictors of the severity of CM so that for each unit increase in depression score, the severity of CM increases by 0.06 units (P = 0.014), and with a one-day increase in the duration of the menstrual cycle, the severity of cyclic mastalgia increases by 0.26 units (P = 0.001) (Table 9).

**Table 9:** Predictors of the severity of cyclic mastalgia.

Predictors	Multiple linear regression					
	B	Beta	T	*p*-value	*95% Confidence Interval for B*	
					Lower	Upper
Depression	0.059	0.132	2.468	**0.014**	0.12	0.105
Duration of Menstrual cycle (day)	0.260	0.172	3.202	**0.001**	0.100	0.419
**R = 0.224**	**R Square = 0.050**			**Adjusted R Square = 0.044**		

## Discussion

Our study demonstrated significant relationships between the severity of cyclic mastalgia (CM) and depression, smoking, and the duration of the menstrual cycle. Regression analysis identified depression and menstrual cycle duration as predictors of CM severity.

We found a positive correlation between depression and CM severity. This aligns with existing literature where depression has been recognized as a correlate of mastalgia [3,4,16,19,22,25], with one study specifically identifying it as a predictor [26]. Biological mechanisms potentially underlying this relationship include: 1) The hypothalamic-pituitary-adrenal (HPA) axis dysregulation affecting gonadal hormone balance [47,48]; 2) serotonin deficiency lowering pain thresholds [49,50]; and 3) Increased prolactin levels due to stress caused by depression [51], which is another factor involved in the development of mastalgia [52].

We observed no significant association between anxiety and CM severity. This finding contrasts with some studies [6,16,26,27] but aligns with others [7]. Yılmaz et al. also found that while anxiety levels are higher in women with mastalgia, increased anxiety does not necessarily lead to increased mastalgia severity [27]. The underlying mechanism may be that both anxiety and depression can lead to an energy imbalance that manifests as mastalgia over time, an uncontrolled activity in the sensory nervous system associated with hormonal imbalances [53]. The inconsistency across studies could be attributed to the use of different measurement tools for anxiety (e.g., state vs. trait anxiety inventories), varying cultural perceptions and reporting of anxiety symptoms, or differences in the comorbidity profile of depression and anxiety within the studied populations. This discrepancy highlights the complex psychosomatic nature of CM and suggests that anxiety and depression may influence mastalgia through distinct pathways. This confirms the importance of including psychological and emotional support in CM treatment [26].

Our analysis revealed a significant positive relationship was observed between menstrual cycle duration and CM severity, with cycle duration also serving as a predictor. This is consistent with a review by Sabery et al. [14], but contrasting with Shobeiri et al. [7]. Given that the menstrual cycle is regulated by complex hormonal interactions critical to mastalgia’s etiology, the link between menstrual patterns and mastalgia appears rational [6]. The longer menstrual cycle, may indicate an underlying hormonal imbalance and prolonged cycles may extend exposure to hormonal fluctuations, exacerbating breast tissue sensitivity [54].

We found no significant correlation between CM severity and menstrual regularity, consistent with Shobeiri et al. [7] but contrasting with reports of positive associations [4,14]. This discrepancy may stem from the distinctive characteristics of our study group, which consisted solely of individuals with severe CM and a low prevalence of regular cycles (6.9%), potentially limiting the variability needed to detect a significant association. Differences in how “regularity” was defined and measured across studies could also contribute to these inconsistent findings. Similarly, we observed no association between dysmenorrhea severity and CM, contradicting studies linking dysmenorrhea to mastalgia [29,55]. The lack of participants with moderate dysmenorrhea in our study may partly explain this divergence.

We identified a significant positive correlation between cigarette smoking and CM severity. This result is confirmed by many other studies [2,3,12,20,21,24]; however, a study report the contrary result [23]. The contrasting result from İdiz et al. could be due to differences in smoking quantification (e.g., pack-years, current vs. former smoker status) or the characteristics of their control group. The potential mechanism is that smoking increases epinephrine, which may contribute to mastalgia [3]; moreover, chronic nicotine exposure can elicit pain hypersensitivity through activation of dopaminergic projections to the anterior cingulate cortex [56].

We found no correlation between body image and the severity of CM. As this relationship has not been explored in previous studies, our findings suggest that body image may not be a significant etiological factor in the development of CM.

Our study revealed no significant relationships between nutritional factors and CM severity, despite controversial findings in existing literature. For instance, while some studies have shown an association between mastalgia and the consumption of caffeine [2–4,20,23,24], salt [19], fast food, dessert, water [23], tea, alcohol [21], chocolate, and fatty diets [2–4], other reports have found no such relationship with caffeine use, alcohol, tea, or a general women’s diet [23,57]. These widespread inconsistencies are likely multifactorial. They may arise from recall bias in dietary assessment tools, variations in the specific types and amounts of foods consumed within broad categories (e.g., different brewing methods for coffee affecting caffeine levels), genetic differences in metabolism (e.g., of caffeine), or the interplay of diet with other lifestyle and cultural factors that were not controlled for in the analyses.

Our findings revealed no relationship between BMI and CM severity, consistent with another Iranian study [7] However, several studies from other populations have demonstrated a link between BMI and mastalgia [4,12,14,19,21,23,58], with findings varying from higher risk in obese individuals (BMI > 30) [19] to higher risk in those with low BMI [59]. These contradictory findings suggest that the relationship between BMI and CM is not linear and may be influenced by factors such as body composition (adipose tissue as a site of estrogen production) and ethnic variations in metabolic health. The average BMI of our participants was 23.27 ± 3.74 kg/m2, with only 5.2% being obese. This limited range may have reduced our power to detect a significant association, should one exist. A study with a wider range of BMI values is needed to fully explore this relationship.

Our analysis showed no significant associations between CM severity and age. Previous studies present conflicting results, with some reporting a positive correlation [7,15] and others a negative one [60]. These discrepancies could reflect differences in the age distribution of the sampled populations (e.g., focusing on younger vs. perimenopausal women) or the influence of cohort effects related to lifestyle factors.

Similarly, we found no relationship between educational level and CM severity, which is a point of contention in the literature; some studies suggest higher education is a risk factor [19,29], while others report the opposite [30]. Education often correlates with other socioeconomic variables; thus, these conflicting results may arise because education is a proxy for different underlying factors (e.g., stress levels, occupational status, health literacy, access to care) in different cultural and healthcare contexts. We also did not find significant correlations with employment or income, though these have been reported elsewhere [28,29].

We found no significant relationship between breast characteristics/breastfeeding history and CM severity. This finding, however, contrasts with several previous studies that have reported a link between mastalgia and specific factors, including breast size size [6,15,61], bra size [19], breastfeeding duration [4], breastfeeding frequency [23,26], benign breast disorders [20,24], a history of malignant breast disease [24], a positive family history of breast cancer [12,29], and a history of previous breast surgery [4]. In a notable conflicting finding, Yigit and colleagues [60] reported that the history of breast cancer was significantly higher in patients without mastalgia. The lack of association in our study could be due to the relatively homogeneous nature of our sample in terms of breast history or the method of data collection for these variables (e.g., self-report vs. clinical examination).

Finally, our study demonstrated no significant relationship between physical activity or sleep duration and CM severity. This contrasts with studies that have confirmed a link between physical activity and a lower incidence of CM [7,22,23] and between a sedentary lifestyle and mastalgia [15,60]. The relationship between physical activity and mastalgia remains a subject of disagreement in the literature [2,12,23,61]. These inconsistencies may be related to the type, intensity, and frequency of physical activity measured, with structured exercise potentially having a different effect than occupational activity. Cultural norms around exercise and sports participation might also influence these results.

Our study showed that only two factors including the duration of the menstrual cycle and depression are predictors of CM severity, which can approve the importance of the effect of hormones regulating the menstrual cycle and related to depression on CM. Most of the studies related to mastalgia presented the relationship of the factors with cyclic and non-cyclic mastalgia together and without separation, while the etiology and the mechanism of pain in these two mastalgia are different. It seems that future studies should focus on cyclic and non-cyclic mastalgia separately. In addition, it is necessary to conduct sampling specifically for each factor or a group of factors, such as only to assess the relationship of nutritional factors on CM, along with measuring the profile of hormones that regulate the menstrual cycle.

### Strengths and limitations

This study has several notable strengths. Primarily, it benefits from a relatively large sample size, which enhances the statistical power and reliability of our findings. Furthermore, the use of valid and reliable standard questionnaires—including Beck’s depression inventory, Spielberger anxiety inventory, Fisher body-image questionnaire, and nominal days with severe pain score (NDBP) scale (Cardiff chart) —strengthens the validity and accuracy of our data collection. Additionally, employing an online questionnaire ensured there were no dropouts and likely facilitated broader participation.

This study has several limitations. Firstly, this was a cross-sectional study, and the results only show correlations between variables; therefore, causal relationships cannot be concluded. To address this, longitudinal studies are suggested for future research. Secondly, the results were based on self-reported information, which is susceptible to biases such as recall bias and social desirability bias, potentially affecting the accuracy of the data. For instance, participants might not accurately remember the intensity of their symptoms or may report them differently due to social desirability.

Another limitation is the cross-sectional assessment of psychological problems including depression and anxiety, which makes it impossible to examine the longitudinal effects of these variables on mastalgia.

Another limitation of this study is the use of female students as the sample. This age and social group may differ from the general female population in terms of factors such as stress levels, lifestyle, and access to educational resources. Consequently, the generalizability of our findings to a broader population may be limited.

For future studies, it is recommended to use more objective methods for data collection. For example, the use of daily symptom diaries could minimize recall bias. Additionally, integrating biological measures (such as hormonal assays) alongside questionnaires could complement self-reported data with objective evidence, thereby enhancing the validity of the findings.

## Conclusion

The present study showed that the severity of CM has a positive correlation with depression, the duration of the menstrual cycle, the duration of menstruation, and smoking. However, only the duration of the menstrual cycle and depression are predictors of CM.

These findings highlight the critical need to integrate mental health screening (particularly for depression) and gynecological assessments into clinical management strategies for CM. From a public health perspective, developing targeted interventions—such as depression management programs, smoking cessation initiatives, and patient education about menstrual health—could significantly reduce the burden of CM and improve quality of life for affected women. Furthermore, future longitudinal studies are needed to conclusively determine the impact of other controversial factors and to explore the complex interplay between hormonal profiles, psychological factors, and pain perception in CM.

## Supporting information

S1 File Mastalgia. Severe.sav. Data. SPSS.
(SAV)

S2 File Mastalgia. Severe. xlsx. Data Excel.
(XLSX)
